# *Agaricus bisporus* Grown on Sustainable Peat Casing Alternatives—A Systematic Review on Quality Characteristics

**DOI:** 10.3390/foods14193348

**Published:** 2025-09-26

**Authors:** Mareike Helena Dissemond, Charlotte Elisabeth Franken, Miriam Sari

**Affiliations:** Competence Center for Applied Mycology and Environmental Studies, Faculty of Food and Nutrition Sciences, Niederrhein University of Applied Sciences, 41065 Mönchengladbach, Germany

**Keywords:** edible mushrooms, peat-free, mushroom quality parameters, nutrients, marketability

## Abstract

Edible mushrooms are increasingly recognized for their high nutritional value and contribution to a healthy diet. Among them, *Agaricus bisporus* is the most commercially important species in Europe and North America. However, the environmental impact of traditional peat use in *A. bisporus* cultivation necessitates the development of sustainable alternatives, given the ecological significance of peatlands. When evaluating casing materials, it is essential to consider not only yield but also other critical factors influencing marketability, such as nutritional value, appearance, and texture. This systematic review examines seventeen studies published between 1989 and 2025 that investigated various peat substitutes while assessing a range of quality criteria. The findings were categorized into seven groups, encompassing both chemical composition and phenotypic characteristics of the fruiting bodies. Most studies focused on the organic and inorganic content of the fruiting bodies, followed by measurements of size and weight. Some alternative casings, for example, increased dry matter contents, which indicates a high solid substance content, such as of proteins or minerals. However, this was not always beneficial, as it could negatively affect texture. Overall, the reviewed studies demonstrate that different casing materials can directly influence quality parameters, and even minor adjustments in casing composition can enhance fruiting body quality.

## 1. Introduction

The importance of edible mushrooms in the human diet is increasing, as they are a valuable source of protein, fiber, vitamins and minerals. The low fat content and bioactive ingredients present in these products make them a significant component of a health-conscious and balanced diet [1].

Five primary genera account for approximately 81% of global mushroom production, with *Agaricus bisporus* (J. E. Lange) Imbach (white button mushroom) accounting for around 11% of the global production volume in the period 2018–2019. *A. bisporus* is the most commercially significant species in Europe and North America [2]. On a global scale, the average consumption of mushrooms is approximately 100 g per individual per week [3]. Atila et al. [4] concluded that the white button mushroom is a nutrient-rich species that contains a multitude of health-promoting ingredients. Its defining characteristics are the significant quantities of proteins, fiber, vitamins (particularly B vitamins and vitamin D) and minerals, such as potassium and phosphorus, as well as a low concentration of fat. Furthermore, *A. bisporus* has been shown to produce bioactive compounds, which have been identified as having antioxidant, immunomodulating and potentially anticarcinogenic properties [4]. One of these compounds comprises β-glucans, which can be found in the cell wall of many basidiomycetes like *A. bisporus.* This distinguishes it not only as a valuable food source but also as a functional food with medicinal potential [1]. Another cell wall component of mushrooms is chitin, which directly affects the texture and therefore the quality of mushroom fruiting bodies [5,6].

### 1.1. The Casing Layer

Mushroom production involves two key phases: a vegetative phase (spawn run) and a reproductive phase (fruiting). Following the inoculation of compost with spawn, the substrate is then subjected to an incubation process under conditions of controlled temperature and humidity. Once fully colonized, the development of fruit bodies of *A. bisporus* requires a non-nutritional layer of casing layer on top of the nutritious compost. The fructification is initiated by lowering air temperature and CO_2_ levels [7].

The casing layer is a key component in mushroom cultivation, providing the physical support necessary for the development of fruiting bodies while simultaneously preventing the underlying compost from drying out [8]. Mushroom cultivation has traditionally relied on peat as casing soil, due to the beneficial properties of peat for enhancing productivity. Peat provides high water-holding capacity, a stable structure, and sufficient porosity, which are all beneficial for mycological growth [9]. However, the extraction of peat raises significant ecological concerns, as it is associated with a substantial ecological footprint and contributes to carbon emissions [10]. Despite covering only approximately 3% of the Earth’s land surface, peatlands store one-third of global soil carbon, thus acting as the second-largest natural carbon sink after the oceans, with the capacity to store up to twice as much carbon as all global biomass. Beyond its function of storing carbon, the importance of peatlands in mitigating floods, preventing erosion, stabilizing climate, reducing drought risk, supplying water to ecosystems, and providing habitats for diverse, often endangered species, is widely established [11]. The environmental significance of peatlands, in conjunction with rising peat prices and more stringent harvesting regulations in leading producing countries such as Canada and Ireland, emphasizes the urgent requirement to establish sustainable alternatives to peat-based casing materials [12].

To successfully replace peat in mushroom cultivation, alternative casing materials must meet a range of essential requirements. They should enable yields and product quality comparable to those achieved with conventional peat-based casings while remaining economically viable and locally available to ensure supply chain stability [13]. Key selection criteria also include environmental sustainability and a reduced ecological footprint. In addition, alternative materials must exhibit consistent physical, chemical, and biological properties to guarantee reliable and reproducible cultivation outcomes [14,15].

A range of alternative substrates are currently under research and investigation for potential use as replacements or supplements to peat in the cultivation of mushrooms. The materials subjected to testing included clay and clayey soils, garden soil, spent mushroom substrate (SMS), and vermicompost (VC). Other examples of such materials are coconut fibers (CF), gravel, sugarcane bagasse, and wood charcoal. Further investigated are compost from rose oil processing (RC), silica gel, tea leaf fibers, wood fibers, composted pine bark, spent coconut husk, tea production waste, and zeolite [16,17,18].

While some of these materials show great potential, others are limited by factors such as natural nutrient content, costs, contamination, water holding capacity, availability, and electrical conductivity. Spent mushroom substrate (SMS), for instance, is the most commonly used alternative casing material because of its high water retention capacity, good availability, and low cost. However, this material does possess certain disadvantages, as it frequently results in increased electrical conductivity, nutrient leaching, and the necessity of an additional composting process prior to utilization [13].

Mushroom growers and casing suppliers worldwide are confronted with several challenges. Increasing environmental pressure and stricter legal regulations suggest the possibility of restrictions or complete bans on the extraction of peat for use in mushroom casings in the long term. This is further complicated by the uncertainty surrounding the agronomic performance of peat-reduced or peat-free alternatives, particularly with regard to yield and quality parameters of the fruiting bodies. Concerns have been raised that alternative casing materials will not perform adequately to maintain economically viable production levels. Furthermore, diminishing peat availability and intensifying competition for bioeconomy materials as biofuel or bio-char will cause shortages and rising prices of alternative casing components [13].

Nevertheless, these alternatives could help mitigate the ecological footprint of mushroom production and promote the recycling of organic waste materials, thereby contributing to a circular economy in agriculture.

### 1.2. Quality Criteria

Cultivated mushrooms in the EU are marketed in accordance with the general marketing standard set out in Delegated Regulation (EU) 2023/2429, Annex I, Part A. In accordance with this regulation, cultivated mushrooms must be intact and healthy. Thus, they must be free from any signs of decay or other defects that could render them unsuitable for consumption. It is important that the items are thoroughly cleaned and virtually free of any visible foreign matter. Furthermore, the mushrooms must be firm, fresh in appearance, practically free from pests and damage caused by pests, and free from abnormal external moisture. It is also necessary for them to be devoid of any foreign odor or taste. The condition of the mushrooms must allow them to survive transport and handling undamaged and arrive at their destination in perfect condition [19].

Special marketing standards are also defined by the United Nations Economic Commission for Europe (UNECE). The UNECE Standard FFV 24 quality differentiation enables categorization into classes Extra, I and II. “Extra”-Class mushrooms must be of superior quality, characterized by well-formed caps and minimal defects. These mushrooms are expected to be nearly free of casing material, ensuring a premium product that meets the highest standards of cultivation and processing. Class I mushrooms are defined by their good quality, with minor deviations in shape, color, or traces of casing material. Class II mushrooms only need to meet minimum standards but may exhibit discolored lamellae and more pronounced defects, provided that shelf life and basic quality are maintained [20].

The classification system is not subject to legal obligation; rather, its function is to provide a description of quality. However, the limit values for pesticide residues in cultivated mushrooms set by the European Commission are legally binding. The contamination of fruiting bodies can occur either through the direct use of pesticides during cultivation or through contaminated substrates. In order to protect consumers, it is essential that these limits are strictly adhered to. Mushrooms absorb and bioaccumulate heavy metals much more rapidly than other agricultural products due to their high accumulation ability [21]. Maximum levels for heavy metals in food are also set by law. For instance, Regulation (EU) 2023/915 establishes maximum levels for cadmium and lead for some cultivated mushrooms [22].

In the USA, the quality of mushrooms is graded by the United States Department of Agriculture (USDA) into U.S. No. 1 and U.S. No. 2. The requirements for fresh mushrooms include the following: they must be of a similar varietal characteristic, mature, fairly well shaped, well-trimmed, and free from open veils, disease, spots, insect injury, decay, and from damage by any cause. The requirements for U.S. No. 2 are equivalent; however, a greater tolerance for open veils and a larger tolerance for defects is permitted [23].

Besides marketing standards or regulatory restrictions, there are also nutritional quality parameters that should not be ignored when producing food.

The aim of this systematic review is to summarize the scientific studies that deal with the cultivation of mushrooms using peat-free or peat-reduced casing materials. The primary focus of this review is to ascertain whether these studies address quality-relevant criteria that are deemed to be pivotal for the commercial viability of the fruiting bodies. The scope of the investigation includes parameters such as nutrient content, weight, color, firmness and texture of the mushrooms, as well as potential contamination and accumulation of harmful substances, with the objective of ensuring the safety of the food and eliminating any potential risks for consumers.

## 2. Materials and Methods

### 2.1. Search Strategy and Database Creation

This systematic review was conducted in strict accordance with the PRISMA protocol [24], ensuring both accuracy and traceability. This included transparent documentation of the search strategy, predefined inclusion and exclusion criteria, a structured screening procedure, and a rigorous assessment of the methodological quality of the selected studies. The literature published in the time period from 1 January 1970 until 15 May 2025 were included and were selected from Web of Science, CAS, CAB Abstracts (only 1970–1989), PubMed, and Springer Nature Link. In an effort to build upon and further the research initiated by Young et al. [25], this study employed their designated search terms:

(mushroom * OR “*Agaricus bisporus*”) AND casing AND peat

NOT (medic * OR clinic *)

NOT (horticulture OR feed OR fodder OR tomato *).

Additionally, the cited literature from Young et al. [25] was searched manually and included as well. Only peer reviewed articles and conference proceedings with their own study results were considered in our search strategy, reviews were excluded.

Following the removal of duplicates, the titles and abstracts of the remaining publications were screened. Availability was checked and reports sought for retrieval were selected. The resulting publications were then checked for relevant backward citations. Publications that were unavailable in English or German were not considered.

The selection criteria employed in this study were based on those outlined by Young et al. [25], with the exception of the critical data, as a more in-depth examination of the quality of the mushrooms was desired. Consequently, the exclusion criterium was applied to all publications that did not incorporate quality parameters. The criteria for inclusion and exclusion are shown in Table 1.

### 2.2. Evaluation of Quality Criteria and Data Extraction

A range of quality criteria were identified in the literature review. The criteria were sorted into two main groups: chemical composition and phenotypic features. The chemical compositions were then sub-grouped into organic and inorganic matter, nutrients, contaminants and heavy metals. The phenotypic features were sub-grouped into size and weight, color, firmness and texture and marketability.

A thorough examination and analysis of the extant literature was conducted with a focus on these characteristics. As the approach to determining quality, as previously outlined, has the capacity to vary, the characteristics were divided into two distinct categories: chemical composition and morphological characteristics. These categories were then subjected to comparative analysis and evaluation.

The data were manually collected and sorted into the two categories by one reviewer and then reviewed by both reviewers. Conflicts were addressed and resolved.

The data were then processed by dividing the two categories into three or four groups, respectively (Table 2). Each thematic category was analyzed and synthesized to identify key differences in the quality criteria of mushrooms grown on alternative casings in the existing literature.

Only results that were statistically significant were included in the visualization of this review.

## 3. Results

### 3.1. Study Selection

The consultation of the databases resulted in the identification of 251 publications, which were added to the 34 publications cited by Young et al. [25], resulting in a total of 285 publications.

**Figure A1:**
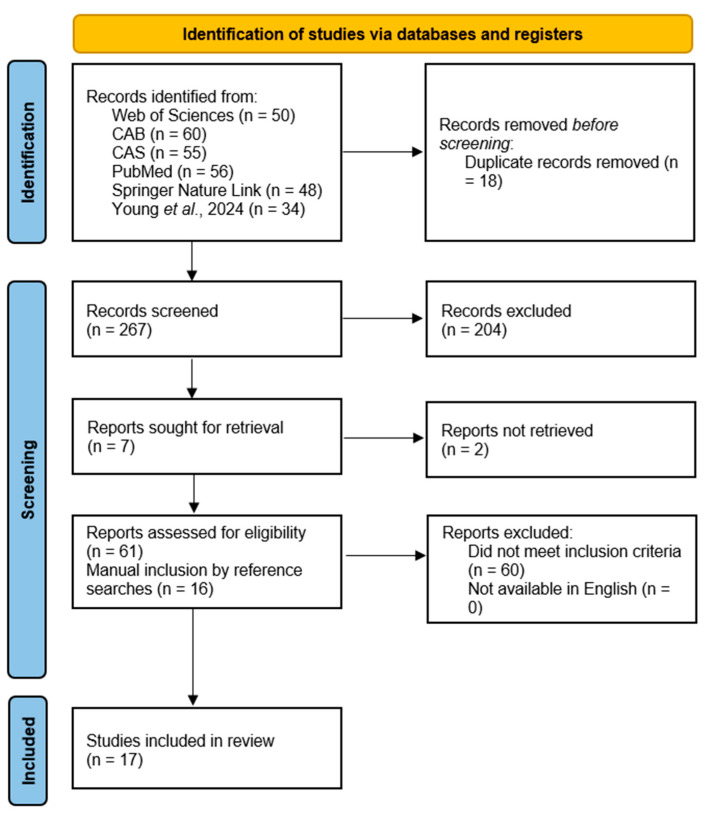
Flow diagram of the literature search and selection phases adapted from the PRISMA guidelines [25].

Following the selection process of the PRISMA methodology (Figure A1) and after the application of the previously outlined criteria (Table 1), 17 publications remained and were selected for further analysis.

### 3.2. Overview of the Identified Studies

In the remaining 17 publications, the authors named 15 different quality parameters that could be sorted into seven groups (Table 2). The analysis of publications concerning the quality parameters of *A. bisporus*-fruiting bodies cultivated on peat substitutes reveals an irregular publication pattern in recent decades.

Although the number of publications published in the 1980s to early 2000s was relatively low, a discernible increase in research activities was observed since 2004, though this declined again in the 2010s. Since 2020, an increasing number of publications have appeared that also examine the quality of fruiting bodies in research on peat substitutes. The year 2021 was identified as the most prolific to date, with three publications.

### 3.3. Peat Substitutes

In total, 18 different peat substitutes that either fully or partwise replaced peat were identified in the found publications. The majority of the used casing materials were made of agricultural side streams or other biodegradable materials. Others were side-streams of the timber industry. Few studies used inert materials, but these were mainly used as partial substitution.

**Figure 1:**
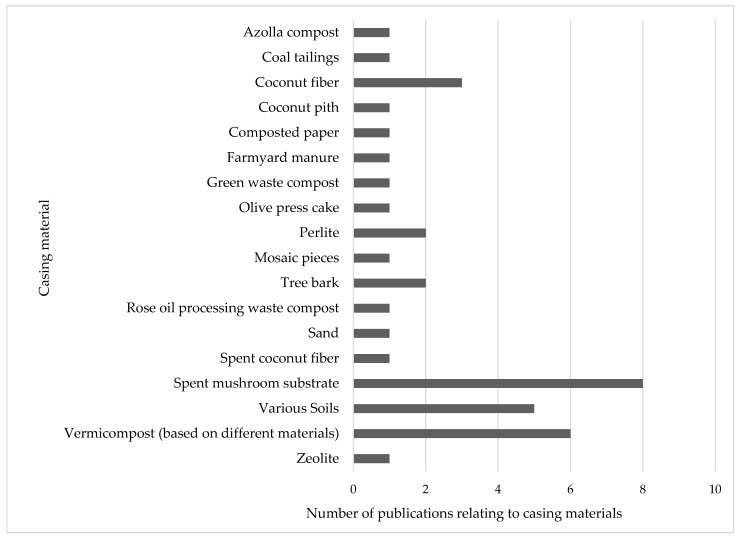
Number of publications in which alternative casing materials were tested.

Spent mushroom substrate, various soils and vermicompost were the most frequently used substitutes. Side-streams from coconut production were also used in many studies. As these varied greatly in their composition, we considered them separately. The following figure details the number of publications that have employed each casing material for the cultivation of *A. bisporus* (Figure 1).

### 3.4. Chemical Composition

#### 3.4.1. Organic and Inorganic Matter

The ash content of the fruiting bodies was determined by ashing the samples at 540 °C in an electric muffle furnace. The dry matter content was determined by drying the samples in an oven until constant weight was achieved.

Cetin et al. [16] reported higher ash contents in the use of olive press cake (OPC) in combination with peat, and even significantly higher contents in the 1:1, 2:1, and 3:1 OPC, plus peat ratios with levels ranging from 12.41 to 13.57 g/100 g (peat: 11.60 g/100 mg).

Pardo et al. [32] compared different *A. bisporus* strains on different alternative casings based on soil and analyzed the ash content of the fruiting bodies as well and highlighted specific differences when using the *A. bisporus* strain Blancochamp BL-40 with a peat casing compared to soil casing, with the ash levels being significantly higher with the peat casing: 117.8 g∙kg^−1^ (soil: 103.6 g∙kg^−1^). But after adding sphagnum peat or black peat to the soil casing, no significant difference can be found.

Askari-Khorasgani et al. [26] noted the highest ash contents of 12.5% with vermicompost from spent mushroom substrate (VSMS) in combination with vermicompost from municipal solid waste (VMSW). However, no significant difference was found compared to the peat control “Duch soil” (11.8%).

Riahi and Zamani’s [36] study revealed that casings composted of a mixture of SMS and azolla compost (AC), with azolla being an aquatic fern, exhibited significantly higher ash (0.80%), organic matter (9.8%), and dry matter (10.7%) contents when compared to conventional peat.

A recent study by Polat and Onel [35] worked with perlite in combination with VC in a solid and liquid state. The casing with the combination of perlite and a dose of 732 g m^−2^ solid VC produced fruiting bodies with the highest dry matter content (7.77%), followed by the combination of perlite and a dose of 3.8 mL m^−2^ liquid VC and 183 g m^−2^ solid VC (7.76%).

Colak et al. [27] investigated various peat casings and forest soil, all also mixed with perlite, sand, or piece of mosaic. They did not observe an increase in the dry matter content of the fruiting bodies when perlite, sand, or pieces of mosaic were added compared to peat (8.50–11.36%), noting either a similar or even lower dry matter content with ranges from 7.9 to 9.96% with the substitutes.

Pardo-Giménez and Pardo-Gonzáles [33], who explored CF and SMS as substitutes for peat, further reported a significantly higher dry matter content in fruiting bodies grown on 100% SMS with 97.4 g kg^−1^. The lowest dry matter content was found in fruiting bodies grown on the combination of mineral soil plus CF (73.2 g kg^−1^), but it was not significantly different to the peat control and 100% CF, or to the combination of CF and SMS in the ration of 4:1.

In another investigation by Pardo-Giménez et al. [34] that assessed the potential of SMS in comparison to peat, significant differences in dry matter content were identified between peat and SMS casing, with dry matter contents of 112.2 g kg^−1^ with a 100% SMS casing and 80.8 g kg^−1^ with the peat casing. However, no significant differences were detected when SMS was mixed with peat, only showing an increase with increasing the SMS content of the casing.

Noble and Dobrovin-Pennington [9] compared various peat casings, which were also substituted with coal tailings, bark, or coir. Their findings indicated that the fruiting bodies’ dry matter content was enhanced by incorporating 25% coal tailings into all casings containing black peat, as well as by adding bark or coir to the black and brown peat casing.

Shandilya [38] found the highest dry matter content in three-year-old-SMS (9.06%), followed by Kashmir peat (8.85%) and a mix of farmyard manure and loam soil (8.55%).

Seaby [14] conducted extensive trials comparing various SMS-based casing. The highest dry matter contents were found with the 100% unsterile and sterile SMS treated with a chelating agent (12.52%, 12.48%), while the lowest dry matter was found with the 100% peat casing (8.14%).

**Figure 2:**
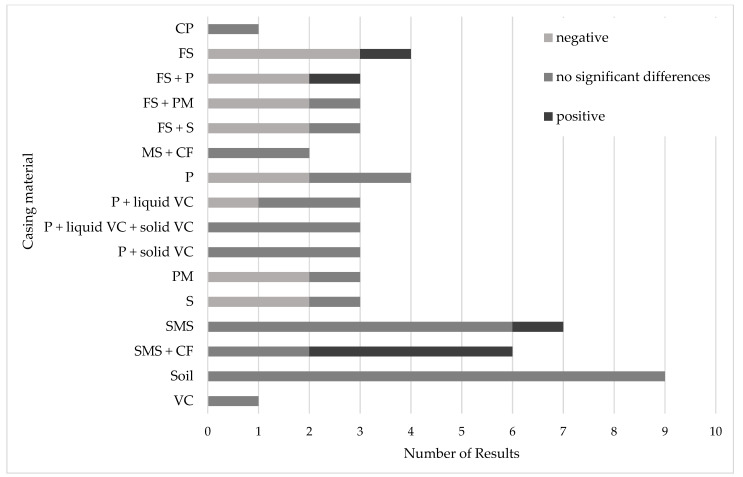
Reported differences in the dry matter content in *A. bisporus* grown on peat-free or peat-reduced casing materials compared to peat control. CF: Coconut fiber pith; CP: Coconut peat; FS: Forest soil; MS: Mineral soil; P: Perlite; PM: Piece of mosaic; S: Sand; SMS: Spent mushroom substrate; VC: Vermicompost.

The following figure illustrates the significant positive and negative results in the dry matter content of *A. bisporus* cultivated on peat-free or peat-reduced casing materials compared to the peat control (Figure 2).

#### 3.4.2. Nutrients

The total nitrogen content was determined using the Kjeldahl method, and the crude protein content was calculated using a fungus-specific conversion factor of 4.38. However, Colak et al. [27] used the general factor of 6.25 for this purpose. The total fat content was evaluated through the use of Soxhlet extraction. The total carbohydrate content was determined by difference calculation, whereby the proportions of protein, fat, water, and ash were subtracted from the total weight.

Cetin et al. [16] highlighted the use of OPC in combination with peat as a casing, documenting significant increases in the nutritional content of the fruiting bodies. They noted that mushroom fruiting bodies grown on the substitutes with OPC plus peat ratios of 1:1, 2:1, and 3:1 caused significantly elevated levels of crude protein (OPC plus peat: 29.04–29.33 g/100 g; peat: 26.86 g/100 g) and crude fat (OPC plus peat: 2.09–2.44 g/100 g; peat: 1.96 g/100 g) compared the peat casing control.

The four OPC plus peat casing variants also showed significantly higher values in the macro- and microelements phosphorus (P), potassium (K), zinc (Zn), manganese (Mn), and copper (Cu) compared to peat. Calcium (Ca), magnesium (Mg) and iron (Fe) values were significantly higher in the peat control.

Riahi and Zamani [36] conducted a study on the effects of SMS and its mixtures with peat and azolla compost, finding in every alternative significantly higher protein values in the fruiting bodies compared to those grown solely on peat, with the highest amounts recorded with SMS alone.

Ghasemi et al. [29] also explored the potential of SMS, as well as coconut peat (CP) and VC, as substitutes for peat casing layers. Their results indicated that while SMS did not significantly alter nitrogen concentration compared to peat, CP showed significantly lower contents ranging between 3 and 4% N in the variants with 2 and 4 cm thick casing layers. VC showed significantly lower nitrogen concentrations only in the 2 cm thick casing layer compared to the 2 cm thick peat casing.

Meanwhile, Colak et al. [27] found in their study that 20% perlite in the different casing bases does not affect the protein content of the fruiting bodies. Further, 20% sand could even increase the protein content of the fruiting bodies in two out of three peat casing and in forest soil (with sand: 21.50–21.81% protein; peat/forest soil: 19.63–20.88% protein). The substitutes either decreased or had no effect on the carbohydrate contents. Only sand increased the contents significantly in one of the three peat casings.

Local soil as a casing alternative to peat was also assessed by Pardo et al. [32]. In their study, they demonstrated that only the *A. bisporus* strain Gurelan 45 had a significantly lower protein content in the peat casing (206.3 g∙kg^−1^) in comparison to a soil–sphagnum peat mixture (235.0 g∙kg^−1^). The *A. bisporus* strains Pla 8.9 and Blanco-champ BL-40 did not show any significant difference in the protein content when grown on different casings.

Further trials by Young et al. [39] involving loamy soil as a casing suggested that inoculation with *Exiguobacterium* sp. and *Bacillus psychrodurans* could elevate polysaccharide contents in fruiting bodies compared to the uninoculated loamy soil, but a direct comparison with peat was not shown.

**Figure 3:**
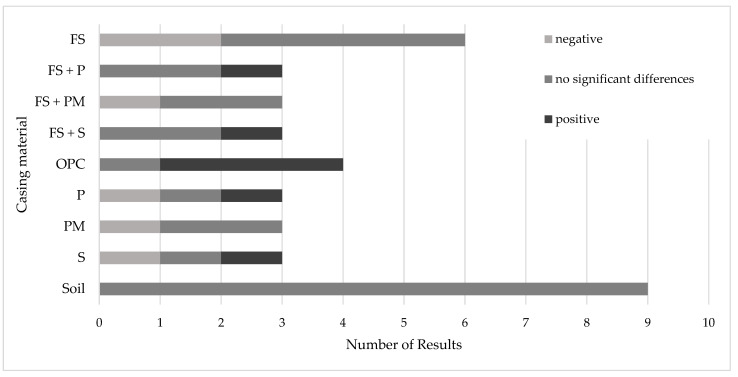
Reported differences in the protein content in *A. bisporus* grown on peat-free or peat-reduced casing materials compared to peat control. FS: Forest soil; OPC: Olive press cake; P: Perlite; PM: Piece of mosaic; S: Sand.

The figure below presents a visual representation of the significant positive and negative results in the protein content of *A. bisporus* cultivated on peat-free or peat-reduced casing materials compared to the peat control (Figure 3).

#### 3.4.3. Contaminants and Heavy Metals

Noble and Dobrovin-Pennington [9] explored whether fine coal tailings lead to significant changes in the heavy metal content of mushroom fruiting bodies. Even with differences in the heavy metal levels in the casings, their studies showed no significant alterations to the heavy metal concentrations in the fruiting bodies.

### 3.5. Phenotypic Features

#### 3.5.1. Size and Weight

Ghasemi et al. [29] noted that the use of CP produced fruiting bodies with the highest cap diameter, with values above 8 cm among all casings tested, and also happened to have the lowest significant yield among all tested casings. The authors showed a negative correlation between cap diameter and yield.

Pardo-Giménez and Pardo-Gonzáles’ [33] findings indicated that while fewer fruiting bodies were produced on 100% SMS, the resultant caps were larger than others, at 35.1 mm. Regarding the coconut casing, the observations of Pardo-Giménez and Pardo-Gonzáles [33] did not align with the observation of Ghasemi et al. [29], since there were no significant differences observed in the quality parameters, cap diameter, or fruiting body size when growing on CF compared to the peat control.

Furthermore, in a more recent study, Duran et al. [28] used RC, spent coconut fiber (SCF), and VC, alongside traditional peat mixtures as casings. Interestingly, they found that a combination of RC and peat (75/25) resulted in fruiting bodies with the largest cap (52.18 mm) and stipe diameters (21.03 mm). With a lower percentage of RC (25%), the highest yield was achieved, with an average cap and stipe diameter as well as a medium average fruiting body weight.

The findings of Pardo-Giménez et al. [34] suggested that fruiting bodies tended to increase in size when cultivated solely on SMS (47.3 mm), but when combined with peat, there was no significant difference in size.

A study by Moctezuma-Perez et al. [31] examined SMS, which was either composted (CSMS) or vermicomposted (VSMS), as a casing. The results revealed that VSMS (50% and 100%) produced the heaviest fruiting bodies (52.61 g, 51.10 g), although with no significant difference when compared to the peat casing (46.56 g). It is noteworthy that the peat substrate also led to mushrooms with the largest cap diameters (6.08 cm), but again without any statistically significant distinctions from the alternatives examined.

Like Moctezuma-Perez et al. [31], Askari-Khorasgani et al. [26] also assessed VSMS. Their findings indicated that the largest mean cap diameter was achieved using a mixture of peat and VSMS (70 mm), while common soil produced mushrooms with the smallest cap diameter (54.3 mm).

Goldwater [30] investigated green waste compost (GWC) as a casing. His results echoed those of previous studies, indicating no significant differences in fruiting body size across the various casings utilized.

**Figure 4:**
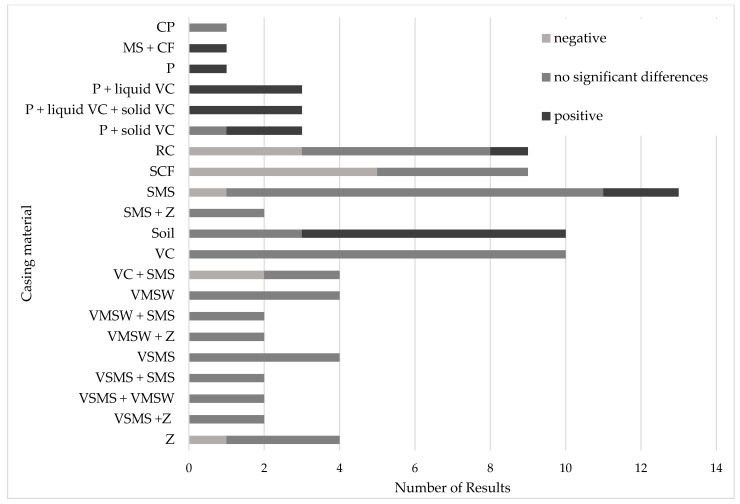
Reported differences in the weight of the fruiting bodies of *A. bisporus* grown on peat-free or peat-reduced casing materials compared to peat control. CF: Coconut fiber pith; CP: Coconut peat; MS: Mineral Soil; P: Perlite; RC: Rose oil processing waste compost; SCF: Spent coconut fiber; SMS: Spent mushroom substrate; VC: Vermicompost; VMSW: Vermicompost from municipal solid waste; VSMS: vermicompost from spent mushroom substrate; Z: Zeolite.

As presented in Figure 4, significant positive and negative results for the weight of the fruiting bodies of *A. bisporus* have been observed when cultivated on peat-free or peat-reduced casing materials. Specifically, soil and perlite, either as sole ingredients or in combination with liquid and/or solid vermicompost, resulted in higher fruiting body weights compared to the peat control.

#### 3.5.2. Color

The color of mushrooms was determined using the CIE Lab* color space, a standardized color representation system comprising three axes: L* is the scale’s lightness dimension, a* is the scale’s green–red spectrum dimension, and b* is the scale’s blue–yellow spectrum dimension. This method enables the precise quantification and description of object color. However, Shandilya [38] did not use the CIE Lab* system. Instead, whiteness was assessed indirectly through the measurement of white light reflection percentage.

The coloration of mushrooms, particularly with regard to their lightness and yellowness values, has been a topic of considerable study in recent years. Various substrates have been tested to determine their impact on these visual parameters, with differing results across the literature.

Pardo et al. [32] found no intrastrain significant differences in the parameters b* and ΔE. In L*, there was only a difference in the strain Gurelan 45, where soil plus black peat (94.1) and a typical peat casing from La Rioja (94.2) were significantly higher than soil alone (93.1) and soil plus sphagnum peat (93.2). The authors suggested that b* and ΔE are not influenced by casing type.

In the trials of Pardo-Giménez and Pardo-Gonzáles [33], the parameter L* showed the lowest values in the casings with 100% CF (92.92) and 100% SMS (92.41), but the mixture of both showed similar values compared to the peat control (93.80). The b*-values showed more significant differences: 25% CF plus 75% SMS (11.246) and 100% SMS (11.088) were significantly higher in b* than the peat control (9.196). Other mixtures did not show any significant difference to the peat casing.

Pardo-Giménez et al. [34] affirmed that *A. bisporus* cultivated solely on SMS produced notably less white fruiting bodies (b*: 12.504) compared to those grown on peat (b*: 8.852). However, when mixed with peat, this disparity was not significant, resulting in relatively equivalent levels of both whiteness and yellowness.

Shandilya [38] conducted a comparative analysis of farmyard manure + loam soil, farmyard manure + tree bark, farmyard manure + SMS, and SMS alone with two different peat casings. The findings revealed that Irish peat casing produced the highest percentage of whiteness: 79.9% reflectance, with the combinations of farmyard manure plus loam soil (74.5% reflectance), followed by farmyard manure plus tree bark (70.1% reflectance). Conversely, mushrooms cultivated in Kashmir peat casing exhibited the lowest whiteness levels (64% reflectance).

Polat and Onel’s [35] results indicated that fruiting bodies grown on perlite accompanied by solid VC (22.8 mL mm^−2^) achieved the highest levels of whiteness (L*: 93.5), subsequently followed by those grown on the combination of perlite plus 7.6 mL m^−2^ liquid and 366 g m^−2^ solid VC (L*: 93.43), as well as those cultivated in peat casing (L*: 93.28).

The study by Duran et al. [28] showed that the combination of 50% SCF and 50% peat had the highest L*-values (92.04), while the significantly lowest L*-values were found for the combination of 75% VC plus 25 % peat (80.95), which was also where the highest values of the parameter b* (10.08) were found. In the second experiment, a lower percentage of VC in peat (40%/60%) achieved the lowest b*-values (7.90) while also having one of the highest L*-values (95.29). This shows how important the right mixture is.

**Figure 5:**
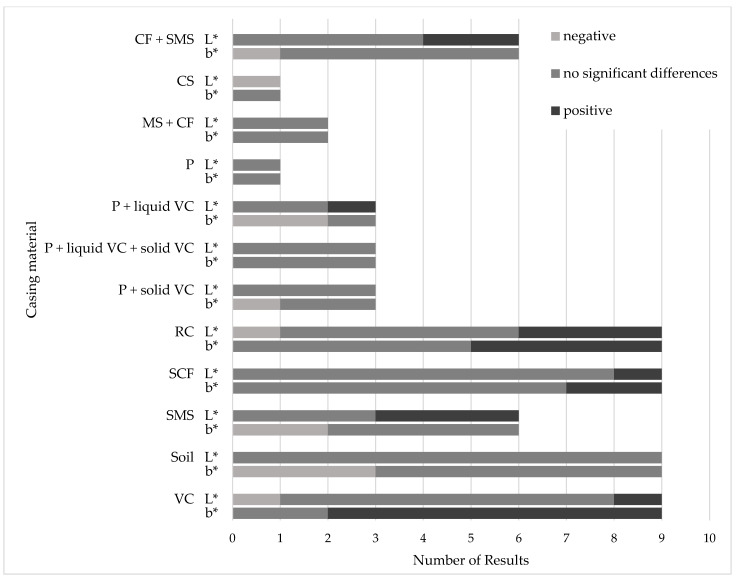
Reported differences in the L*- and b*-values of *A. bisporus* grown on peat-free or peat-reduced casing materials compared to peat control. CF: Coconut fiber pith; CS: Casing soil; MS: Mineral soil; P: Perlite; RC: Rose oil processing waste compost; SCF: Spent coconut fiber; SMS: Spent mushroom substrate; VC: Vermicompost.

The following figure displays the significant positive and negative results observed in the L*- and b*-values of *A. bisporus* cultivated on peat-free or peat-reduced casing materials in comparison to the peat control (Figure 5).

#### 3.5.3. Firmness and Texture

The firmness of the mushrooms was determined by employing puncture or compression tests. In these tests, pressure is applied to the center of the cap or the cut mushroom stems with a cylindrical stamp or needle of a defined size and speed until a break or penetration point is reached. The force required for this action can be used to assess the texture or firmness of the fruiting body. However, it should be noted that Seaby [14] employed a subjective assessment to evaluate the texture of the mushrooms.

In the recent study by Duran et al. [28], the highest cap firmness of mushrooms was observed when 75% RC in the first experiment (2.22 kg/cm^2^) and 50% RC in the second experiment (3.87 kg/cm^2^) was combined with peat. Conversely, the lowest firmness was recorded in the peat casing (1.42 kg/cm^2^) during the first experiment, while in the second experiment, a combination of 50% SCF and 50% peat (2.40 kg/cm^2^) produced the least firm caps.

Pardo-Giménez and Pardo-Gonzáles [33] reported a significantly higher firmness in mushrooms cultivated on 100% SMS, with a puncture force of 30.2 N, a finding that was also reflected in the dry matter content of the fruiting bodies, where the same casing variant achieved the highest dry matter content. The lowest firmness measured with a puncture force of 19.8 N was found in the combination of mineral soil plus CF, which was handled as one of two controls. The lowest firmness measured by a compression energy of 86.3 mJ was found with 100% CF. Further reinforcing this notion, Pardo-Giménez et al. [34] discovered a significantly higher firmness when high amounts of SMS (75% SMS/25% peat) (30.5 N) were utilized as casing, or when SMS was used alone (33.5 N). However, they noted no significant differences to the peat control with smaller amounts of SMS.

Pardo et al. [32] found no intrastrain differences with the strains Pla 8.9 and Gurelan 45 in the parameter firmness. Only the strain Blancochamp BL-40 showed a significantly higher firmness when grown with soil as a casing (22.5 N) compared to soil plus sphagnum peat (20.3 N) and the La Rioja casing type (18.2 N), but there was no significant difference compared to soil plus black peat (21.8 N). With each strain, the lowest firmness of the fruiting body was found with the peat casing from La Rioja.

Seaby’s [14] findings indicated that fruiting bodies exhibiting low dry matter tended to be pink, become easily bruised, and to open at a small size, while those with high dry matter were tougher, chewier, and developed into larger closed-cup mushrooms. The optimum texture was found to be associated with a dry matter content of approximately 7 to 8%. It is interesting to note that only the 100% peat casing produced mushrooms with a dry matter content of 8.14%, while all other casing types resulted in higher dry matter levels. It is noteworthy that the 100% unsterile SMS treated with a chelating agent exhibited the highest dry matter content at 12.52%, which correlated with the poorest texture.

#### 3.5.4. Marketability

During the harvesting process, Pardo et al. [32] evaluated and segregated fruiting bodies that did not conform to marketable standards regarding size, color, or disease-free conditions, but they did not further explain in detail why individual fruiting bodies were described as non-marketable. In their trials, no significant intrastrain differences in the numbers of non-marketable fruiting bodies were identified between the various casings.

In the experiments of Polat and Onel [35], only the peat control scored below 90% marketable yield (62.92%), perlite plus 366 g m^−2^ solid VC (96%), and perlite plus 732 g m^−2^ VC (99.61%) were also below 100%, but not significantly lower. Marketable yield was achieved when no abnormalities in size or weight occurred, but the authors also did not further explain in detail why individual fruiting bodies were described as non-marketable. The fruiting body weight in the peat control was also significantly lower than the rest, except for the perlite plus 732 g m^−2^ solid VC casing.

In the study of Sassine et al. [37], the outcomes of solely composting paper without the addition of any supplements resulted in fruiting bodies of non-marketable quality. The fruiting bodies that were observed were noted to be malformed and exhibited significantly slower growth rates when compared to those cultivated with peat as the casing. Fruiting bodies grown on the composting paper without any supplements were found to be firmer, larger, and in smaller numbers in comparison to peat.

## 4. Discussion

### 4.1. Chemical Composition

The selection of substrates and casing materials plays a crucial role in determining the quality and nutritional composition of cultivated mushrooms. As shown in the results, the emerging evidence indicates that alternative casing materials, including olive oil processings, soil and various organic composts, can successfully enhance both yield and the chemical composition in mushroom production.

The chemical composition of the fruiting bodies of *A. bisporus* is of particular importance, as mushrooms are considered an important source of nutrients that promote good health. For instance, they encompass all nine amino acids that are essential for humans, rendering them a valuable source of protein [40]. It is imperative to ensure that the nutrients present in the fruiting bodies remain consistent, as a reduction in these nutrients would result in a reduction in quality.

It is essential to analyze the dry matter content, defined as the sum of all solid substances like proteins, minerals and chitin, as this provides direct information regarding the nutrient status or the physical quality of the fruiting body. Further information regarding mineral substances can be obtained through the determination of ash content, given the fact that minerals are incapable of combustion and therefore cannot be burnt.

Dry matter content was strongly affected by the choice of the casing material. Organic -rich casings such as SMS and VC often resulted in higher dry matter contents [33,34,35,36,38]. Riahi and Zamani’s [36] study also demonstrated that elevated levels of dry matter were accompanied by augmented protein concentrations in fruiting bodies cultivated on SMS-based casings. Meanwhile, inert substitutes like sand or perlite did not significantly improve the dry matter content and occasionally resulted in lower values than the peat control [27]. It is interesting to note that, despite the presence of similar or lower levels of dry matter in comparison to the control groups, an increase in protein concentration was also observed within the sand casing. This suggests that modifying the casing may lead to alterations in nutrients concentrations. But it is important to note that a decrease in dry matter does not necessarily imply a comprehensive reduction in all solid substances.

Furthermore, dry matter can also be an important indicator of texture and firmness, as dry matter also determines components of the fungal cell walls, such as chitin [1]. However, no paper has investigated whether the casing had an effect on the chitin concentration, which represents a major gap in the literature, since chitin is a key structural polysaccharide influencing the texture and may vary in response to changes in the nutrient availability provided by different casing materials.

Increased ash contents in fruiting bodies grown on casings enriched with organic residues, such as OPC, SMS, and other composts, suggest a higher overall mineral uptake. These elevated ash values reflect enhanced mineral accumulation and may be indicative of improved nutritional density. For instance, Cetin et al. [16] reported ash contents in the combination of OPC and peat higher than these seen in the peat control. Similar findings were reported by Riahi and Zamani [36] and Askari-Khorasgani et al. [26].

Cetin et al. [16], Riahi and Zamani [36] and Colak et al. [27] all found higher protein levels in some of their substitutes, while others found no significant differences or even lower contents [29,32].

Higher protein levels in the fruiting bodies can be explained by a higher protein or nitrogen content in the casing soil [16,36]. However, it is important to note that for these nutrients to be absorbed by the fungus, they must be bioavailable. To illustrate this point, the findings of Ghasemi et al. [29] show that the highest values in the casing do not automatically convert into the highest values in the fruiting body. The researchers identified elevated nitrogen levels in VC, yet their observations did not reveal a parallel distribution within the fruiting bodies. This contrast can be observed once more in the coconut peat variant, where the second-highest nitrogen concentrations were identified, yet the lowest values of nitrogen were found in the fruiting bodies [29].

Ghasemi et al. [29] and Riahi and Zamani [36] both analyzed fruiting bodies grown on SMS, but Ghasemi et al. [29] did not reproduce the higher protein levels that were found in Riahi and Zamani [36] trials. While the nitrogen concentrations, measured by Ghasemi et al. [29], remained comparable to those found in peat and were not significantly lower, and it is evident that organic substrates such as SMS exhibit considerable variance, with fluctuations manifesting in diverse forms. Several factors could contribute to these variations, including differences in the composition of SMS and the specific strains of *A. bisporus* used.

Pardo et al. [32] showed how important the choice of the strain is: the strain Gurelan 45 had significantly lower protein values than Blancochamp BL-40 on the same casing.

Besides SMS, soil was also often used as a casing: Young et al. [39] used loamy soil, Pardo et al. [32] used local soil, and Colak et al. [27] used forest soil. However, beyond their utilization of the term “soil”, these casing alternatives bear little similarity and can therefore not be compared. Both Young et al. [39] and Colak et al. [27] tried to improve their substitutes with different additives. While Young et al. [39] inoculated bacteria naturally occurring in *Agaricus* sp. cultivation to their substitute to enhance productivity and nutrient contents, Colak et al. [27] incorporated perlite, sand and pieces of mosaic into their substitute, showing that both perlite or sand can improve chemical compositions of the fruiting bodies, like a higher protein concentration, when added to the forest soil compared to forest soil alone, but also to three out of four peat casings [27].

Besides positive chemical compositions, it is necessary to analyze residues of contaminants in the fruiting body as well, since mushrooms are able to accumulate contaminants like heavy metals and can also be used for remediation [41]. Unfortunately, only Noble and Dobrovin-Pennington [9] conducted an analysis for heavy metals. The absence of literature on this subject indicates a significant area for future research, particularly in light of the potential health risks associated with the consumption of high quantities of heavy metals, emphasizing the necessity to observe regulatory limits. For example, the acceptable limit for Cd is 0.05 mg/kg of fresh matter, while for Pb, the acceptable limit is 0.3 mg/kg of fresh matter [22].

It is imperative to note that the analysis of pesticide residues was not conducted in any of the reviewed studies. This critical absence of analysis is particularly concerning, as the presence of residues that exceed maximum residue limits has the potential to pose significant health risks to consumers. Furthermore, such residues may also have consequences for the marketability and regulatory compliance of the final product. Given that many of the proposed casing substitutes originate from agricultural side-streams, which may retain chemical residues or contaminants that mushrooms are able to accumulate, rigorous testing is essential to ensure the safety and suitability of these substitutes for their use in the food production.

In summary, the choice of substrate and casing material significantly influences the organic and inorganic matter of mushrooms. Innovative mixtures and alternative materials show promising results in enhancing certain nutrient compositions and the overall quality of fruiting bodies. Future research should aim to further optimize these substrate combinations to improve yields and the nutritional quality in mushroom cultivation, without increasing any kind of harmful residues.

### 4.2. Phenotypic Features

The extant literature demonstrates that the utilization of peat-reduced or peat-free casing materials in the cultivation of *A. bisporus* is associated with a variety of effects on yield and fruiting body quality. A notable divergence has emerged between the yield quantity and the fruit body size and weight.

Ghasemi et al. [29] demonstrated that the utilization of CP resulted in the development of notably large fruiting bodies. However, it was observed that CP exhibited the lowest yield among all the casing materials that were examined. A similar tendency was previously observed by Pardo-Giménez and Pardo-Gonzáles [33]: the utilization of 100% SMS resulted in a reduced yield of fruiting bodies, yet these exhibited a substantial size in comparison to alternative casings. The findings of this study indicate that peat-reduced casings frequently necessitate a compromise between yield quantity and fruiting body quality. However, Ghasemi et al. [29] emphasize that, given the consideration of ecological and economic aspects, particularly in the context of SC for cost reduction and waste recycling, a minor decline in yield can be considered tolerable. In contrast, however, the results of Duran et al. [28] demonstrate that a mixture with 25% RC not only achieved the highest yield, but also resulted in fruiting bodies with average cap and stem diameters and medium weight. A mixture containing 75% RC was previously observed to result in the largest cap and stalk diameters. The findings emphasize the significance of a targeted mixture design in optimizing both qualitative and quantitative parameters.

SMS deserves particular consideration as an alternative casing material. A multitude of studies have demonstrated the potential of this medium to produce comparable fruiting body qualities, with or without the addition of peat. The utilization of pretreatment methods such as composting or vermicomposting has been demonstrated to enhance the quality of SMS as a casing material. These processes have the capacity to reduce the electrical conductivity and nutrient content of the substrate [42]. Consequently, these conditions are considered conducive to the optimal development of mushroom growth [43], while simultaneously minimizing the potential for undesirable microbial activity [8]. Pardo-Giménez et al. [34] observed a significant increase in fruiting body size when cultivated on 100% SMS, although these differences were no longer significant when combined with peat. As Moctezuma-Pérez et al. [31] also reported, VSMS produced the heaviest fruiting bodies, with no significant difference observed when compared to pure peat. Askari-Khorasgani et al. [26] also confirmed the effectiveness of VSMS: a mixture of peat and VSMS resulted in the largest average cap diameters, while normal soil produced significantly smaller fruiting bodies. The findings demonstrate that SMS, particularly VSMS, provides a reliable foundation for peat-reduced casings. However, the effect appears to be strongly dependent on the type of pretreatment and the percentage of the latter in the substrate.

Finally, a number of studies suggest that materials reduced in peat content may be equivalent in terms of their properties. In the study conducted by Moctezuma- Pérez et al. [31], no statistically significant variations were observed in the dimensions or mass of fruiting bodies between peat-free (SMS, VSMS) and peat-containing casing. In addition, Goldwater [30] concluded that no significant differences in fruiting body size could be detected when different casing materials were employed. These findings indicate the practical applicability of peat-free or peat-reduced alternatives. Despite the absence of substantial enhancements in performance outcomes, comparable results to the established peat variant are still consistently demonstrated. Consequently, the potential for sustainable substrate alternatives in the cultivation of mushrooms is significant.

The UNECE Standard FFV 24 [20] has defined size categories for cultivated mushrooms based on the diameter of the cap. These categories are small (15–45 mm), medium (30–65 mm) and large (>50 mm). A review of the extant literature indicates that even peat-reduced cover soils can facilitate the cultivation of mushrooms that meet the standards of the UNECE Standard FFV 24 [20], and in some cases, exceed the average size. It has been demonstrated that mixtures containing vermicomposted materials or RC have a high potential for the formation of large fruiting bodies. Conversely, the exclusive utilization of individual alternative materials, such as SMS, is frequently associated with a diminished cap diameter, which often results in the cultivation of mushrooms of smaller to medium-sized dimensions. The selection and integration of alternative components, therefore, assumes a pivotal role in ensuring conformity with market-relevant size standards.

The color quality of the mushrooms is an important marketing factor, as it is directly associated with the freshness and quality of the fruiting bodies at the time of purchase. In this particular context, the L*- and b*- values of the fruiting bodies were analyzed in detail.

A number of studies have demonstrated that the utilization of complete peat substitutes frequently results in a decrease in brightness and an increase in yellow coloration. For instance, the studies conducted by Pardo-Giménez and Pardo-Gonzáles [33] and Pardo-Giménez et al. [34] demonstrated that 100% SMS or CF resulted in significantly lower L*-values and higher b*-values in comparison to conventional peat. These disparities could be substantially mitigated by appropriate combinations with peat, resulting in a lack of statistical significance. A more pronounced differentiation of the color results is demonstrated in the study by Duran et al. [28]. In this study, a significant reduction in the L*-value and an increase in the b*-value were observed, suggesting a shift towards a more pronounced green hue. This outcome was attributed to the incorporation of a substantial proportion of VC, constituting 75% of the total sample. Furthermore, a mixture containing 40% VC and 60% peat was found to yield both high brightness and low yellow coloration. The findings demonstrate that even minor alterations in the mixing ratio can result in substantial impacts on color perception.

In contrast, Pardo et al. [32] reported no significant differences between the tested casings for certain parameters such as b* and ΔE, which suggests a certain variety de-pendency. Nevertheless, a significant difference in the L*-value was also found in this study for one of the tested strains (Gurelan 45), with casings containing black and Sphagnum peat showing higher brightness values than pure soil casings.

A comparison with the ideal values for color rendering defined by Uccello et al. [44] (L* = 96, b* = 8, ΔE < 5) shows that these are rarely achieved, especially without complete or high replacement of peat. Whilst certain peat-reduced mixtures have been shown to achieve L*-values exceeding 93 in some cases [28,35], a significant proportion of variants, including those consisting of 100% SMS or coconut fiber, have been observed to exhibit substantially lower b*-values, frequently exceeding the targeted level of 8, thereby indicating a visible yellow coloration. As posited by Uccello et al. [44], the mixture of pigments with a b*-value greater than 10 and an L*-value less than 94 is likely to result in a ΔE-value greater than 8, thereby signifying a substantial reduction in optical quality. Consequently, many peat-free variants exhibit visual disadvantages in comparison to conventional substrates. These findings once again highlight the need to evaluate peat-free casings in a differentiated manner according to mixture, preparation, and substrate structure, rather than evaluating them across the board.

Texture is a key quality characteristic of edible mushrooms, as it has a significant influence on sensory perception and marketability. Consequently, the objective measurement of firmness constitutes an essential component of quality control during cultivation.

A number of studies have demonstrated that the utilization of SMS as a casing material has resulted in an increase in the firmness of the fruiting bodies. For instance, Pardo-Giménez et al. [34] demonstrated a substantial enhancement in strength when utilizing 100% and 75% SMS. In a similar context, Pardo-Giménez and Pardo-Gonzáles [33] reported that 100% SMS not only achieved the highest firmness but also the highest dry matter content of the fruiting bodies (9.47%). As stated previously, the dry matter content can be an indicator of texture and firmness due to its role in constructing the fungal cell walls [1].

However, these results contradict the findings of Seaby [14], who demonstrated that elevated levels of dry matter (>8%) were associated with adverse sensory characteristics. In this study, the optimal perception of texture was attained with a dry matter content of approximately 7–8%, a level that was exclusively achieved with a 100% peat casing. This finding suggests that an increase in firmness does not necessarily lead to an improvement in sensory quality.

It is notable that alternative substitute materials demonstrate divergent behavior. Pardo-Giménez and Pardo-González [33] found that combinations of mineral soil with CF and pure CF resulted in the lowest measured strength values, as determined by both the puncture test and the compression energy. Duran et al. [28] also observed this trend, finding that the lowest firmness was achieved with a mixture of 50% SCF. Conversely, RC emerged as a promising material: in both series of experiments conducted in this study, casings containing 75% and 50% RC attained the highest strength values for the fruiting bodies.

The sum of all already named parameters can be summarized under the term marketability, as the critical quality parameters directly influence commercial viability. Despite its importance, the criteria used to classify mushroom fruiting bodies as marketable or non-marketable were often insufficiently detailed in the reviewed studies. For example, Pardo et al. [32] excluded fruiting bodies based on general deviations in size, color, or disease symptoms but did not define specific thresholds. No significant differences in the proportion of non-marketable specimens were found among the various casings, suggesting limited casing influence under their trial conditions. In contrast, Polat and Onel [35] reported effects that were more pronounced and surprising. The peat control produced the lowest percentage of marketable fruiting bodies (62.92%), significantly lower than all other treatments. Although marketable yield was defined as the absence of abnormalities in size or weight, the authors did not clarify what constituted such abnormalities. One explanation for the high percentage of non-marketable yield could be the significantly reduced fruiting body weight. Sassine et al. [37] observed severe morphological issues in their studies as well and therefore excluded the data from their article.

International standards provide structured criteria for the assessment of fruiting body quality. These categories, designated as Extra, I, and II, are based on shape, cleanliness, presence of casing residues, and surface defects [20]. Although not legally binding, these standards offer important guidance for interpreting marketability. Within this framework, many of the non-marketable specimens reported in the literature could likely be classified in lower quality categories due to shape irregularities, bruising, or visible casing residues—attributes potentially influenced by casing composition.

It is recommended that future research efforts be directed towards the classification of fruiting bodies into these categories, with a subsequent explanation provided to clarify the rationale behind this categorization.

The integration of additional databases could have resulted in a more substantial number of relevant studies; however, the accessibility of publications was remarkably limited, even within the utilized databases. Consequently, a large proportion of publications remained inaccessible.

## 5. Conclusions

Selecting an appropriate casing material is crucial for the quality of cultivated mushrooms. As this review illustrates, casing materials affect both the chemical composition and phenotypic features of fruiting bodies. Additionally, the optimal proportion and formulation of casing materials can dramatically alter cultivation outcomes. Even minor adjustments or mixing ratios have been shown to improve the fruiting body quality, underscoring the need for precise, evidence-based optimization.

A significant research gap exists in the analysis of contaminants, such as heavy metal- and pesticide-residues in the fruiting bodies. Standardized testing is crucial to ensure food safety, especially since many alternative casing materials are derived from agricultural or industrial by-products, that may contain these residues.

The comparison of peat casing alternatives is also challenging, since differences in experimental conditions, measurement methods, substrate composition and *A. bisporus* strains can strongly influence results, as shown in this review. To better understand the range of available materials, consistent research designs and evaluation criteria are essential.

From a bioeconomic perspective, it is also important to take ecological sustainability and economic profitability into consideration. Political decisions and targeted subsidies can play a guiding role by promoting the use of alternative casing soils. As such materials become more widely accepted and distributed, it is expected that their production costs will fall, making them more competitive in terms of price.

To summarize, the existing literature demonstrates that peat casing alternatives have the capacity to both maintain and enhance the yield, nutritional quality and sensory attributes of *A. bisporus* fruiting bodies. It is recommended that future research seek to enhance the optimization of these casings with the goal of ensuring the successful production of high-quality food, whilst simultaneously reducing the economic consequences of peat use.

## Figures and Tables

**Table 1:** Criteria for inclusion and exclusion of papers in dataset for the analysis.

Category	Inclusion	Exclusion
Comparator	Control group using peat casing	Absence of a control group
Study Design	Experimental, with controlled growth trials	Study design without replicates
Species	*Agaricus* sp. with no restriction based on variety or size at harvest	Edible or medicinal fungal species that are not members of the genus *Agaricus*
Experiment	Mushrooms grown using peat alternative casing. This can either be a partial or complete substitution, with no limitation on precise composition	Mushrooms grown exclusively on peat
Data	The observation of at least one quality parameter was undertaken (e.g., fruiting body color, nutrients of the fruiting body, marketable size of the fruiting body)	Quality parameters of the fruiting body were not addressed

The identification and selection process of the studies was conducted by two reviewers and is summarized in the flow diagram (Figure A1).

**Table 2:** Quality criteria that are used in the identified studies.

References	Organic/ Inorganic Matter	Nutrients	Contaminants/Heavy Metal	Size/Weight	Color	Firmness/ Texture	Marketability
Askari-Khorasgani et al. (2015) [26]	X			X			
Cetin et al. (2025) [16]	X	X					
Colak et al. (2007) [27]	X	X					
Duran et al. (2023) [28]				X	X	X	
Ghasemi et al. (2020) [29]		X		X			
Goldwater (2021) [30]				X			
Moctezuma-Pérez et al. (2021) [31]				X			
Noble and Dobrovin-Pennington (2005) [9]	X		X				
Pardo et al. (2010) [32]	X	X		X	X	X	X
Pardo-Giménez and Pardo-Gonzáles (2008) [33]	X			X	X	X	
Pardo-Gimenéz et al. (2011) [34]	X			X	X	X	
Polat and Onel (2021) [35]	X			X	X		X
Riahi and Zamani (2008) [36]	X	X					
Sassine et al. (2005) [37]							X
Seaby (1999) [14]	X						
Shandilya (1989) [38]	X			X	X		
Young et al. (2012) [39]		X					
Number of publications.	11	6	1	10	6	4	3

## Data Availability

The data that support the findings of this study are available from the corresponding author, Miriam Sari, upon reasonable request.

## Abbreviations

The following abbreviations are used in this manuscript:

*A. bisporus*	*Agaricus bisporus*
Ca	Calcium
CF	Coconut fiber pith
CP	Coconut peat
CSMS	Spent mushroom substrate compost
Cu	Copper
Fe	Iron
GWC	Green waste compost
K	Potassium
Mg	Magnesium
Mn	Manganese
OPC	Olive press cake
P	Phosphorus
RC	Rose oil processing waste compost
SCF	Spent coconut fiber
SMS	Spent mushroom substrate
UNECE	United Nations Economic Commission for Europe
VC	Vermicompost
VSMS	Spent mushroom substrate vermicompost
VMSW	Municipal solid waste vermicompost
Zn	Zinc
